# Age-related changes in static balance in older women aged in their early sixties to their late eighties: different aging patterns in the anterior–posterior and mediolateral directions

**DOI:** 10.3389/fnagi.2024.1361244

**Published:** 2024-04-08

**Authors:** Shun Sasagawa, Ai Arakawa, Aimi Furuyama, Yasuo Matsumoto

**Affiliations:** Department of Human Sciences, Faculty of Human Sciences, Kanagawa University, Yokohama, Japan

**Keywords:** posture, quiet standing, center of pressure, fall, women

## Abstract

**Objective:**

The aim of this study was to cross-sectionally investigate how static balance changes throughout the aging process in older women aged from their early sixties to their late eighties.

**Methods:**

Forty-six older women (aged 62–89 years) were requested to stand barefoot and quietly on a force platform for 30 s with their eyes either open or closed. During the trials, the position of the center of foot pressure (CoP) and the acceleration of the body’s center of mass (ACC) were measured. The root mean square (RMS) of the CoP and ACC values was calculated to evaluate the amplitude of postural sway and the level of regulatory activity, respectively. The mean power frequency of the ACC was also calculated to represent the temporal characteristics of regulatory activity.

**Results:**

In the anterior–posterior direction, there was no significant relationship between the RMS of CoP and the participants’ age, whereas the RMS of ACC significantly increased with increasing age. In the mediolateral direction, however, the RMS of CoP significantly increased with increasing age, whereas the RMS of ACC did not change with age. The mean power frequency of ACC did not exhibit any age-related change in either the anterior–posterior or the mediolateral direction.

**Conclusion:**

The results indicate that static balance in older women aged in their early sixties to their late eighties exhibits distinctly contrasting aging patterns between the anterior–posterior and mediolateral directions. To prevent falls in older women, it is necessary to elucidate the physiological mechanisms responsible for the increase in mediolateral sway that occurs throughout old age.

## 1 Introduction

For more than 15 years, Japan has been a super-aged society: in 2021, 28.9% of the total Japanese population was aged 65 years and older (Japanese Cabinet Office, 2022). In an aging society, falls among older people are a major health problem. According to a survey by the Tokyo Fire Department (2020), fall accidents accounted for more than 80% of emergency transport cases of older people, of which 40% were diagnosed as moderate or severe cases requiring hospitalisation. Even if falling does not result in severe injury, the fear of falling can decrease activities of daily living of older people, thereby creating a vicious cycle that increases the risk of falls (Scheffer et al., 2008). Notably, older women have been reported to be at higher risk of hip fracture (Farmer et al., 1984) and are more likely to develop a fear of falling after experiencing a fall (Vellas et al., 1997) than men. Therefore, to prevent falls and to improve the quality of life of older people, especially of older women, it is important to advance the current understanding of aging effects on their balance abilities. Because the number of emergency transport cases per unit age of population has been reported to increase dramatically as age increased from 65 to 95 years (Tokyo Fire Department, 2020), it is likely that even in old age, balance abilities change throughout the aging process and these changes are closely related to risk of fall in older people. Therefore, to prevent falls of older people, it is especially important to investigate age-related changes in static balance throughout old age. Although numerous attempts have been made to compare the balance abilities of older individuals to those of younger people (see Quijoux et al., 2021 for a review), a few attempts have been made to examine the age-related changes in balance throughout old age. Recently, Brech et al. (2022), who evaluated the age-related changes on static balance in 400 women aged 50–89 years, have reported that the older women in their eighties exhibited greater displacement and range of the center of foot pressure (CoP) in the mediolateral (ML) direction and faster planar mean velocity of CoP than the women in their fifties and sixties. However, because Brech et al. (2022) have examined only displacement-related measures (i.e., displacement and range of CoP) for each of the anterior–posterior (AP) and ML directions, direction-dependency of the age-related changes in static balance is still ambiguous. Furthermore, because Brech et al. (2022) have not examined any of frequency-domain measures, age-related changes in the temporal aspects of static balance remains subject to debate. Therefore, in the present study, our aim was to cross-sectionally investigate how static balance in older women aged in their early sixties to their late eighties changes throughout the aging process, in both the AP and ML directions, using multiple measures (i.e., displacement-related measure, acceleration-related measure, and frequency-related measure) that enabled us to capture a wide range of spatio-temporal features of postural control.

Static balance ability is usually assessed using the CoP time series obtained using a force platform measurement. When analyzing the CoP time series, it should be noted that it contains information on both the position and acceleration of the center of body mass (CoM) (Kuo et al., 1998; Zatsiorsky and Duarte, 2000); positional fluctuations of the CoM are reflected in low-frequency CoP sway below 0.5 Hz, whereas fluctuations of CoM acceleration are reflected in high-frequency sway above 0.5 Hz (Zatsiorsky and Duarte, 2000). The information on CoM position and CoM acceleration have different meanings in the control of upright balance. The amplitude of positional fluctuations of the CoM represents the level of stability achieved by the postural control system (Hufschmidt et al., 1980), which can be evaluated by displacement-related sway measures such as the root mean square (RMS) of the CoP (Maurer and Peterka, 2005). In contrast, the amplitude of CoM acceleration describes the level of regulatory activity (Hufschmidt et al., 1980), which can be characterised by velocity-related sway measures such as the mean velocity of the CoP (Masani et al., 2014). The CoM acceleration is not only indirectly estimable from the CoP time series; it can also be directly obtained from the horizontal ground reaction force according to Newton’s Second Law of Motion (McClenaghan et al., 1996; Masani et al., 2007; Yu et al., 2008; Sasagawa et al., 2009; Oba et al., 2015). This directly obtained CoM acceleration (ACC) has been suggested to be an alternative force platform measure for assessing changes in the postural control system. In fact, recent studies have reported that the ACC has a higher sensitivity to changes in the postural control system with development (Oba et al., 2015), aging (McClenaghan et al., 1996; Masani et al., 2007) and disease (Yu et al., 2008). In this study, we therefore investigated the effects of aging on different aspects of static balance using measurement of both the CoP and the ACC.

## 2 Materials and methods

### 2.1 Participants

To determine the sample size needed for the study, we conducted an *a priori* power analysis in G∗power using a bivariate normal model from the exact family with a two-tailed test. Correlation ρ (H_1_), α error probability, power (1 – β error probability) and correlation ρ (H_0_) were set to 0.40, 0.05, 0.80 and 0.00, respectively. The correlation ρ (H_1_) of 0.4 was based on a previous study that showed a correlation coefficient of 0.42 between the age of older participants and the sway amplitude in the ML direction (Lord et al., 1999). The required minimum sample size was calculated to be 46. Participants were recruited through a flyer distributed throughout the local area. The inclusion/exclusion criteria to participate in the study were older women who could stand for a few minutes without standing aids. To provide an opportunity of checking their balance abilities for community-dwelling older women, we did not use strict inclusion/exclusion criteria to participate in the study. Eighty-four independent community-dwelling women aged between 54 and 89 years participated in the study. They gave written informed consent after receiving a detailed explanation of the study. The procedures used in this study were in accordance with the Declaration of Helsinki and were approved by the institutional ethics committee (no. 2021–34). For the analysis, we excluded the data of participants who: (1) were under 60 years old (*N* = 2); (2) had a reported history of neurological disorders (*N* = 2); or (3) were receiving treatment for neurological or musculoskeletal disorders (*N* = 18). We also excluded the data of participants such as who could not accomplish the entire trial due to fatigue, who made voluntary movements during the trials, or who wore tights or stockings (*N* = 16). Eventually, data from 46 individuals 62–89 years old (mean ± standard deviation (SD): age 79.2 ± 6.1 years, height 151.4 ± 5.7 cm, body mass 50.4 ± 7.2 kg) were used in the analysis.

### 2.2 Procedure and measurements

The participants stood barefoot and quietly on a force platform (TFG-4060; Tec Gihan, Kyoto, Japan) with their feet parallel to each other (heel-to-heel distance of 20 cm) and with their arms at their sides. They stood with their eyes open (EO) for 30 s, then for 30 s with their eyes closed (EC); this was repeated three times. During the EO condition, the participants were asked to gaze at a target placed at eye level 2.5 meters in front of them. The ground reaction force data were sampled at 100 Hz through an analog-digital converter (PowerLab 16/35, ADInstruments, Sydney, Australia) for offline analysis.

### 2.3 Data processing and analysis

**Figure 1:**
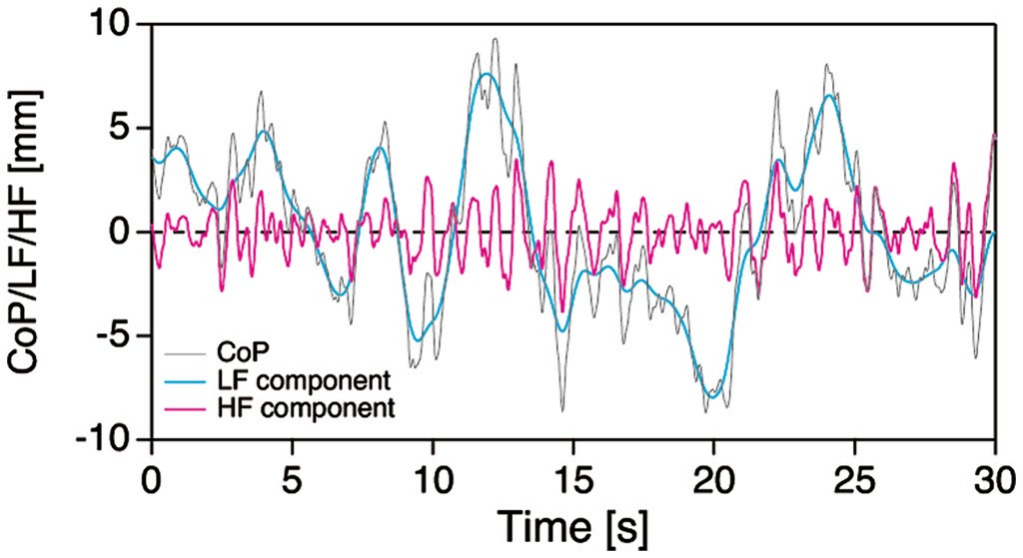
Typical example of decomposition of the CoP sway (grey line) into the LF (cyan line) and HF (magenta line) components. CoP, center of foot pressure; HF, high frequency; LF, low frequency.

From the measured ground reaction forces, the CoP position in the AP and ML directions were calculated for each participant. We also calculated the ACC in both the AP and ML directions by dividing the horizontal ground reaction forces by the participants’ body mass (Masani et al., 2007; Yu et al., 2008; Sasagawa et al., 2009; Oba et al., 2015). The CoP and ACC time series were digitally smoothed using a fourth-order, dual-pass, low-pass Butterworth filter (MATLAB signal processing toolbox, MathWorks, Natick, MA, United States) with a cut-off frequency of 5.0 Hz (Prieto et al., 1996). The fluctuations of the CoP and ACC were evaluated by the RMS. The RMS of CoP (CoP_RMS_) represents the amplitude of postural sway, or the effectiveness of postural control (Hufschmidt et al., 1980). In contrast, the RMS of ACC (ACC_RMS_) describes the level of regulatory activity generated by the postural control system (Hufschmidt et al., 1980; Morasso and Schieppati, 1999). In the analysis, we also performed decomposition of the CoP sway into their low-frequency (LF) and high-frequency (HF) components (Figure 1). The LF component was obtained by smoothing the raw CoP time series with a cut-off frequency of 0.5 Hz (Caron et al., 1997; Loram and Lakie, 2002; Yamada-Yanagawa et al., 2022). By subtracting the LF component from the low-pass filtered CoP time series, we then obtained the HF component.

For the CoP, ACC, LF component and HF component time series, we performed spectral analyses (MATLAB signal processing toolbox, MathWorks, Natick, MA, United States). The data for a single 30-s trial were detrended and then divided into five segments with 50% overlap (10 s of data in each segment). A fast Fourier transform algorithm was used for each segment to calculate the power spectral density after passing it through a Hamming window, which resulted in a frequency resolution of 0.1 Hz. The power spectral density function of the individual segments was ensemble-averaged into the function of a single trial. The temporal characteristics of these time series were quantified by the mean power frequency (MPF).

### 2.4 Statistical analysis

The average value across three 30-s trials was used as a representative value of the EC and EO condition for each participant. In the text and tables, data are presented as mean ± SD. To allow comparison of the data regardless of differences in body size, we performed normalisation on the RMS of CoP-and ACC-related variables by dividing the RMS by the participants’ body height (BH). Before comparing each variable, the normality of the data was verified with the Kolmogorov–Smirnov test. The Kolmogorov–Smirnov test showed that all variables were normal. Pearson correlation coefficients were calculated to examine the relationship between the participants’ age and CoP-or ACC-related variables. An absolute correlation coefficient < 0.20 was considered as very weak, that between 0.20–0.39 as weak, that between 0.40–0.59 as moderate, that between 0.60–0.79 as strong, and that >0.80 as very strong (Evans, 1996). A raw *p* < 0.05 and a Benjamini–Hochberg false discovery rate (Benjamini and Hochberg, 1995) of <15% were used to indicate statistical significance. A false discovery rate threshold of 15% was chosen to avoid a type II error. In the text, tables and figures, raw *p*-values are reported.

## 3 Results

**Figure 2:**
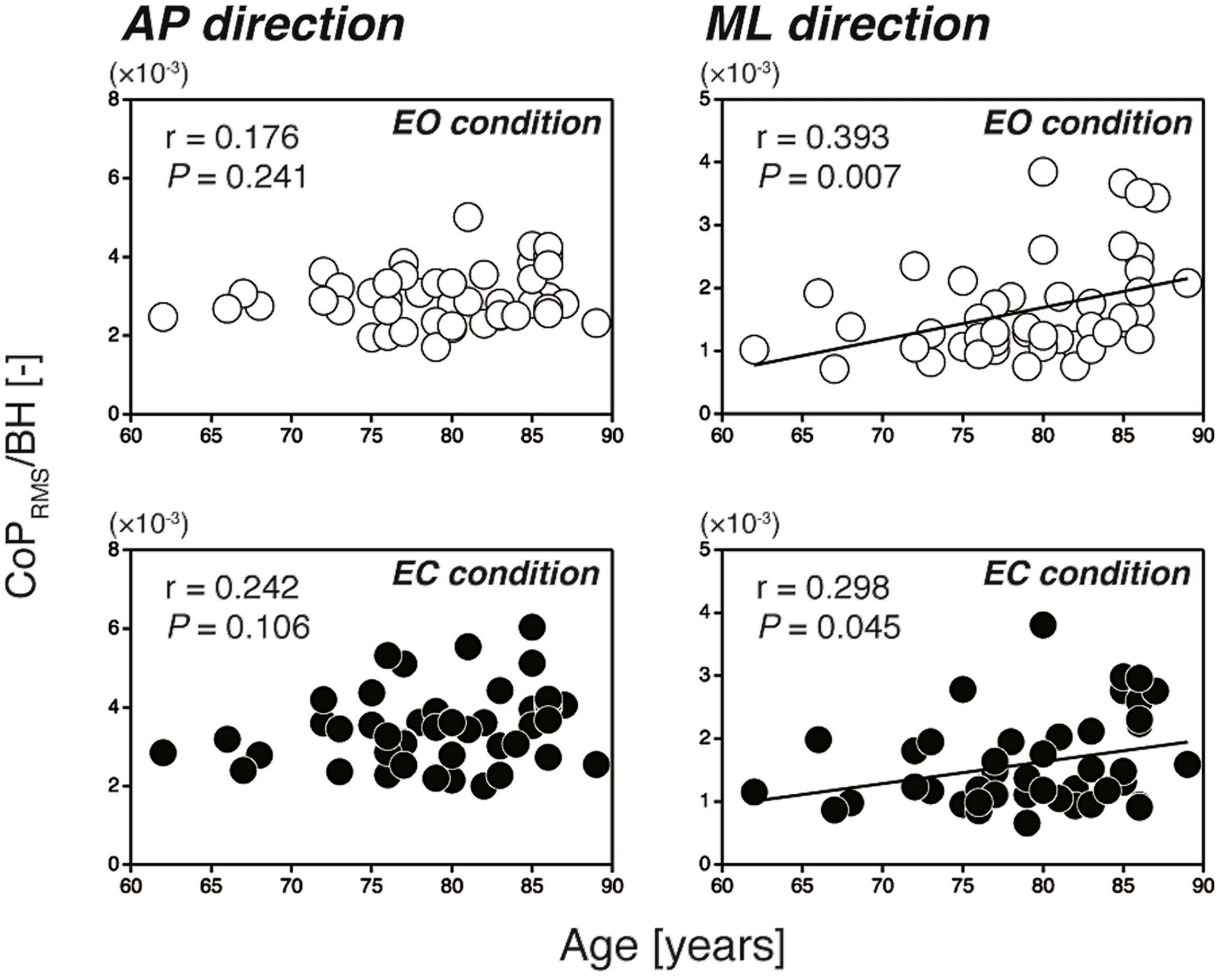
Scatter plots of the CoP_RMS_ against the participants’ age (left-hand panels; AP direction, right-hand panels; ML direction). The open and filled circles indicate the EO and EC conditions, respectively. In the AP direction, the CoP_RMS_ had no significant correlation with age (EO: *r* = 0.176, *p* = 0.241; EC: *r* = 0.242, *p* = 0.106). In the ML direction, the CoP_RMS_ had a significant positive correlation with age (EO: *r* = 0.393, *p* = 0.007; EC: *r* = 0.298, *p* = 0.045). AP, anterior–posterior; BH, body height; CoP_RMS_, amplitude of postural sway; EC, eyes open; EO, eyes closed; ML, mediolateral.

**Figure 3:**
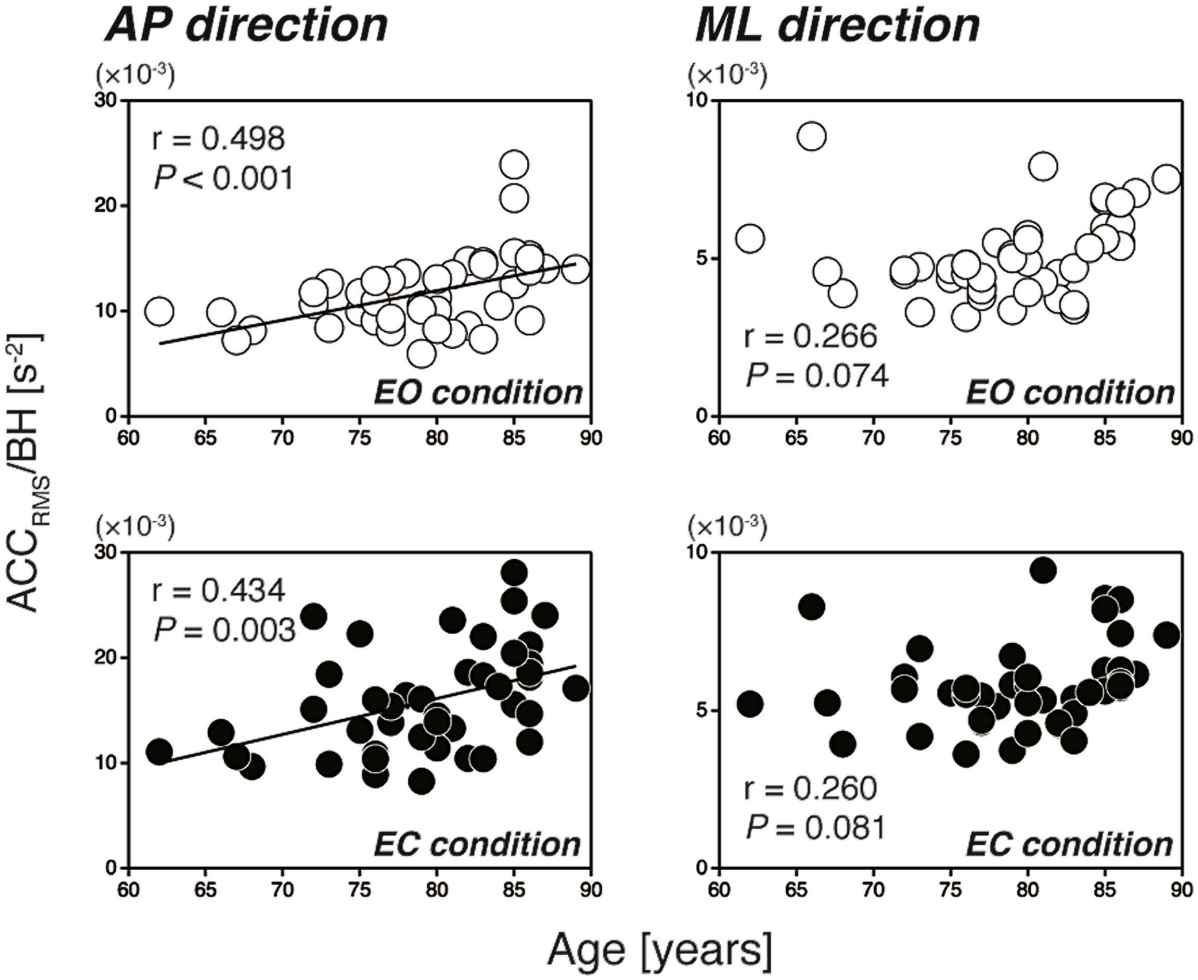
Scatter plots of the ACC_RMS_ against the participants’ age (left-hand panels: AP direction, right-hand panels: ML direction). The open and filled circles indicate the EO and EC conditions, respectively. In the AP direction, the ACC_RMS_ had a significant positive correlation with age (EO: *r* = 0.498, *p* < 0.001; EC: *r* = 0.434, *p* = 0.003). In the ML direction, the ACC_RMS_ had no significant correlation with age (EO: *r* = 0.266, *p* = 0.074; EC: *r* = 0.260, *p* = 0.081). ACC_RMS_, level of regulatory activity; AP, anterior–posterior; BH, body height; EC, eyes open; EO, eyes closed; ML, mediolateral.

Figure 2 shows the scatter plots of the CoP_RMS_ against the participants’ age for all participants (*N* = 46). In the AP direction, the CoP_RMS_ had no significant correlation with age during either the EO or EC condition (EO: *r* = 0.176, *p* = 0.241; EC: *r* = 0.242, *p* = 0.106). In contrast, in the ML direction, the CoP_RMS_ had a significant weak positive correlation with age during both the EO and EC conditions (EO: *r* = 0.393, *p* = 0.007; EC: *r* = 0.298, *p* = 0.045). The reverse was evident when the relationships between the participants’ age and the ACC_RMS_ were examined, as shown in Figure 3. In the AP direction, the ACC_RMS_ had a significant moderate positive correlation with age during both the EO and EC conditions (EO: *r* = 0.498, *p* < 0.001; EC: *r* = 0.434, *p* = 0.003). In contrast, in the ML direction, the ACC_RMS_ had no significant correlation with age during either the EO or EC condition (EO: *r* = 0.266, *p* = 0.074; EC: *r* = 0.260, *p* = 0.081).

In the AP direction, the MPF of CoP (CoP_MPF_) had a significant weak positive correlation with the participants’ age (EO only: *r* = 0.308, *p* = 0.037) (Table 1). In contrast, in the ML direction, the CoP_MPF_ had a significant weak negative correlation with age (EO: *r* = −0.394, *p* = 0.007; EC: *r* = −0.299, *p* = 0.044) (Table 1). A naïve interpretation of these results might lead to the conclusion that, in the AP direction, the frequency of regulatory activity increased with increasing age, whereas, in the ML direction, the frequency decreased with age. However, this is not necessarily true. In fact, the MPF of ACC (ACC_MPF_) had no significant correlation with age in either the AP or ML direction (Table 1). The mechanisms of significant age-related changes in the CoP_MPF_ can be explained if the CoP sway is decomposed into the LF and HF components before considering the age-related changes in their amplitude (i.e., the RMS) and frequency (i.e., the MPF). From Table 1, we can see that the MPF of both the LF and HF components had no significant correlation with age in either the AP or ML direction. In the AP direction, however, the RMS of the HF component had a significant weak-to-moderate positive correlation with age (EO: *r* = 0.423, *p* = 0.003; EC: *r* = 0.395, *p* = 0.007) (Table 2). In contrast, in the ML direction, the RMS of the LF component had a significant weak-to-moderate positive correlation with age (EO: *r* = 0.425, *p* = 0.003; EC: *r* = 0.323, *p* = 0.029) (Table 2). In summing up, the LF and HF components of the CoP sway did not change their temporal characteristics with age in either direction. However, the LF component in the ML direction and the HF component in the AP direction slightly increased their amplitude with increasing age. Thus, with advancing age, the LF component in the ML direction and the HF component in the AP direction increased their relative dominance in the frequency spectrum of the CoP sway, thereby resulting in lower CoP_MPF_ and higher CoP_MPF_ values in the ML and AP directions, respectively.

**Table 1:** Summary of the MPF of CoP-and ACC-related variables and their correlations with the participants’ age.

		EO condition		EC condition	
		MPF [Hz]	Correlation	MPF [Hz]	Correlation
AP direction	CoP	0.365 ± 0.098	0.308*	0.435 ± 0.114	0.219
	ACC	1.093 ± 0.282	−0.111	1.131 ± 0.268	0.061
	LF	0.194 ± 0.041	0.264	0.215 ± 0.042	0.236
	HF	1.173 ± 0.156	0.098	1.251 ± 0.210	−0.045
ML direction	CoP	0.389 ± 0.098	−0.394**	0.444 ± 0.141	−0.299*
	ACC	0.960 ± 0.174	−0.126	0.969 ± 0.178	−0.025
	LF	0.223 ± 0.050	−0.233	0.236 ± 0.055	−0.082
	HF	1.036 ± 0.097	−0.083	1.077 ± 0.126	−0.023

^*^p < 0.05; ^**^p < 0.01. Data are presented as mean ± SD. ACC, acceleration of centre of mass; AP, anterior–posterior; CoP, center of foot pressure; EC, eyes open; EO, eyes closed; ML, mediolateral; MPF, mean power frequency; SD, standard deviation.

**Table 2:** Summary of the RMS of LF and HF components and their correlations with the participants’ age.

		EO condition		EC condition	
		RMS/BH [−] (×10^−3^)	Correlation	RMS/BH [−] (×10^−3^)	Correlation
AP direction	LF	2.738 ± 0.742	0.091	3.055 ± 0.916	0.234
	HF	0.849 ± 0.292	0.424**	1.228 ± 0.415	0.395**
ML direction	LF	1.587 ± 0.876	0.425**	1.497 ± 0.776	0.323*
	HF	0.409 ± 0.151	0.158	0.476 ± 0.163	0.180

^*^p < 0.05; ^**^p < 0.01. Data are presented as mean ± SD. AP, anterior–posterior; BH, body height; EC, eyes open; EO, eyes closed; HF, high frequency; LF, low frequency; ML, mediolateral; RMS, root mean square; SD, standard deviation.

## 4 Discussion

The results showed that, in the AP direction, the amplitude of postural sway (measured by the CoP_RMS_) had no significant correlation with the participants’ age (Figure 2, left-hand panels), whereas the amplitude of regulatory activity (measured by the ACC_RMS_) had a significant moderate positive correlation with age (Figure 3, left-hand panels). Our findings are in line with previous studies comparing static balance between younger and older adults (Prieto et al., 1996; Masani et al., 2007; Kouzaki and Masani, 2012). For example, Masani et al. (2007) demonstrated that there was no statistical difference in the SD of CoP between the younger (29.0 ± 7.7 years) and older (72.7 ± 5.6 years) adult groups, whereas there was a significant difference in the SD of ACC between the two groups. The findings of this and other previous studies suggest that, in the AP direction, the postural control system in older individuals maintains the amplitude of postural sway within a certain range by increasing the level of regulatory activity. In other words, the level of regulatory activity associated with a certain level of stability may increase with advancing age.

In the ML direction, the sway amplitude had a significant weak positive correlation with age (Figure 2, right-hand panels), whereas the amplitude of regulatory activity did not have a significant correlation with age (Figure 3, right-hand panels). This slightly increased sway amplitude with increasing age in the ML direction is consistent with the recent report of Brech et al. (2022) in which statistically significant difference was found in the ML directional CoP displacement between the women in their sixties and eighties. However, our present results are partly contrary to a previous study (Prieto et al., 1996) in which no significant differences were found in any of ten measures based on the ML directional component of the CoP (including the CoP_RMS_) between the younger (26.4 ± 4.9 years) and older (68.0 ± 1.3 years) adult groups. The discrepancy between the previous (Prieto et al., 1996) and present studies may be attributed partly to the difference in the age of the participants; in the present study they were substantially older (79.2 ± 6.1 years) than the participants of Prieto et al. (1996). This discrepancy, at the same time, suggests that the CoP_RMS_ in the ML direction may increase steeply after 70 years of age. The slightly increased sway amplitude together with unchanged levels of regulatory activity in the ML direction further suggests that the postural control system is not sensitive to the sway amplitude in this direction, which is in marked contrast to the control in the AP direction. Researchers (Day et al., 1993; Winter et al., 1996) have pointed out that the balance controls in the AP and ML directions are completely different processes involving two independent neuromuscular groups. This difference in the balance control between the AP and ML directions is due largely to a difference in the structure of the human body between the sagittal and frontal planes. In particular, independent motion is possible at the ankle, knee and hip joints in the sagittal plane, whereas this is not possible in the frontal plane because of the mechanical linkage of the ankle and hip joints (Day et al., 1993). Furthermore, it is important to note that a loss of balance in the ML direction can more easily lead to a fall because the loaded supporting leg is in the direction of fall (Winter et al., 1996). In fact, it has been shown that the ML directional CoP_RMS_ in a blindfolded condition was the best predictor of future fall risk in older people (Maki et al., 1994). Other studies have also demonstrated that the ML directional sway in narrow stance was increased in older people with a history of falls (Lord et al., 1999; Melzer et al., 2004). Combined with the findings of the previous studies (i.e., relationship between the ML directional sway and the falling risk of older people) and that of our present study (i.e., slightly increased ML directional sway with increasing age), we are now confident that the changes in ML balance occurring throughout old age can provide an insight into the increasing incidence of falls with age.

The results further showed that the CoP_MPF_ had a significant weak positive correlation with the participants’ age in the AP direction, whereas the CoP_MPF_ had a significant weak negative correlation with age in the ML direction (Table 1). However, this result does not mean that the frequency of regulatory activity changes with advancing age. Rather, the changes in CoP_MPF_ observed in old age result from the marginal increase in the amplitude (but not frequency) of the LF (in the ML direction) and HF (in the AP direction) components (Table 2). In fact, the ACC_MPF_ had no significant correlation with age in either the AP or ML direction (Table 1). Nevertheless, in the AP direction, the ACC_MPF_ of the older participants (1.112 ± 0.274 Hz, the data from the EO and EC conditions were averaged) tended to be lower than those of young adults (1.3 ± 0.2 Hz) and children (1.6 ± 0.2 Hz) reported in Oba et al. (2015) [note that the ACC_MPF_ values reported in Oba et al., 2015 were also obtained by averaging the data from eyes-open and eyes-closed conditions]. The difference in ACC_MPF_ in the AP direction between the older participants and younger ones is presumably due to the redundancy in the multi-joint dynamics inherent in the sagittal plane as discussed above. When the human upright stance in the sagittal plane is modelled as a double-link inverted pendulum consisting of the leg and head–arms–trunk segments (Sasagawa et al., 2009, 2014), there are two coordination modes between these segments known as the in-phase and anti-phase coordination modes (Suzuki et al., 2012). The in-phase coordination mode, in which the leg and head–arms–trunk segments move in the same direction, has an eigenfrequency of below 0.5 Hz (Günther et al., 2009, 2011; Suzuki et al., 2012, 2016; Morasso et al., 2019). In contrast, the anti-phase coordination mode, in which the two segments move in opposite directions, has an eigenfrequency of around 1.0 Hz (Günther et al., 2009, 2011; Suzuki et al., 2012, 2016; Morasso et al., 2019). Therefore, the lower ACC_MPF_ seen in the older participants suggests that the in-phase coordination mode increases its relative dominance in controlling upright balance, and thus these individuals sway more like a single-link inverted pendulum rotating around the ankle joint during quiet standing.

In conclusion, we found that static balance in older women aged from their early sixties to their late eighties exhibits distinctly contrasting aging patterns between the AP and ML directions; i.e., an unchanged sway amplitude with an increased level of regulatory activity in the AP direction, and a slightly increased sway amplitude with an unchanged level of regulatory activity in the ML direction. Based on the literature and our present findings, we suggest that to prevent falls in older women, it is necessary to elucidate physiological mechanisms responsible for the increase in the ML sway throughout old age.

## Data availability statement

The original contributions presented in the study are included in the article/supplementary material, further inquiries can be directed to the corresponding author.

## Ethics statement

The studies involving humans were approved by The Ethics Committee of Kanagawa University. The studies were conducted in accordance with the local legislation and institutional requirements. The participants provided their written informed consent to participate in this study.

## Author contributions

SS: Conceptualization, Data curation, Formal analysis, Funding acquisition, Investigation, Methodology, Resources, Software, Supervision, Validation, Visualization, Writing – original draft, Writing – review & editing. AA: Data curation, Formal analysis, Investigation, Validation, Writing – original draft, Writing – review & editing. AF: Data curation, Formal analysis, Investigation, Validation, Writing – original draft, Writing – review & editing. YM: Conceptualization, Data curation, Funding acquisition, Investigation, Project administration, Resources, Supervision, Writing – review & editing.
